# Epidermal growth factor receptor inhibitor-related skin toxicities: a review of management and possible preventive and therapeutic approaches for Asian patients by the Japanese Pharmacist-led Oncodermatology Study Team

**DOI:** 10.1007/s10147-025-02868-1

**Published:** 2025-09-01

**Authors:** Yohei Iimura, Hirotoshi Iihara, Yoshitaka Saito, Hisanaga Nomura, Takuya Iwamoto, Mayumi Kotera, Yusuke Tsuchiya, Tatsuya Sumiya, Mariko Kono, Daisuke Hirate, Tomohiro Kurokawa, Toshinobu Hayashi, Hironobu Hashimoto, Junichi Higuchi, Ryuta Urakawa, Hiroyuki Saotome, Seiichiro Kuroda

**Affiliations:** 1 Department of Pharmacy, The IMSUT Hospital, The Institute of Medical Science, The University of Tokyo, 4-6-1Minato-Ku, Shirokanedai, Tokyo 108-8639 Japan; 2 Department of Pharmacy, Gifu University Hospital, Gifu, Japan; 3 Department of Clinical Pharmaceutics & Therapeutics, Faculty of Pharmaceutical Sciences, Hokkaido University of Science, Sapporo, Japan; 4 Department of Clinical Pharmacology and Therapeutics, Kyoto University Hospital, Kyoto, Japan; 5 Department of Pharmacy, Mie University Hospital, Tsu, Japan; 6 Department of Pharmacy, Ageo Central General Hospital, Saitama, Japan; 7 Department of Pharmacy, Yokohama City Minato Red Cross Hospital, Yokohama, Japan; 8 Department of Pharmacy, Tokyo Metropolitan Police Hospital, Tokyo, Japan; 9 Department of Pharmacy, Teine Keijinkai Hospital, Tokyo, Japan; 10 Department of Surgery, Jyoban Hospital of Tokiwa Foundation, Fukushima, Japan; 11 Department of Medical Epigenomics Research, Fukushima Medical University, Fukushima, Japan; 12 Department of Emergency and Disaster Medical Pharmacy, Faculty of Pharmaceutical Sciences, Fukuoka University, Fukuoka, Japan; 13 Department of Pharmacy, National Cancer Center Hospital, Tokyo, Japan; 14 Department of Pharmacy, Ohara Healthcare Foundation Kurashiki Central Hospital, Okayama, Japan; 15 Department of Pharmacy, The University of Osaka Dental Hospital, Osaka, Japan; 16 Department of Clinical Pharmacy Research and Education, Graduate School of Pharmaceutical Sciences, The University of Osaka, Osaka, Japan; 17 Department of Pharmacy, Jyoban Hospital, Public Interest Foundation Tokiwakai, Fukushima, Japan

**Keywords:** EGFR inhibitor, Acneiform rash, Paronychia, Pruritus, Supportive care, Asia

## Abstract

Epidermal growth factor receptor antibodies and tyrosine kinase inhibitors cause various skin toxicities. Acneiform rash, paronychia, and pruritus are the major side effects, and their incidence is high, especially in Asian patients. These skin disorders greatly reduce the patients’ quality of life and can affect treatment intensity. As the incidence and severity of these skin toxicities correlate with treatment effects, adequate management during the treatment period is essential. Guidelines and treatment recommendations exist for epidermal growth factor receptor inhibitor-related skin toxicities. However, there have been no previous reviews of studies on Asian patients. In this review, we discuss the possible preventive and therapeutic recommendations for Asian patients. We derived recommendations based on evidence from Asian patients. This review will contribute to the management of these toxicities in Asian populations.

## Introduction

Epidermal growth factor receptor (EGFR) antibodies (panitumumab and cetuximab) and EGFR tyrosine kinase inhibitors (TKIs; gefitinib, erlotinib, afatinib, osimertinib, and dacomitinib) cause various skin disorders. For example, the incidences of acneiform skin rash, paronychia, dry skin, and pruritus caused by these treatments are relatively high. These side effects can decrease patient quality of life (QOL) [1] [2] and lead to treatment discontinuation [3]. Furthermore, secondary infections can be caused by the affected site [4]. Approximately 80–90% of patients receiving EGFR inhibitors experience skin toxicities [5]. Therefore, discussions on the management of skin toxicities in all patients treated with EGFR inhibitors are essential. Because the incidence and severity of EGFR inhibitor-related skin toxicities correlate with treatment outcomes [6–8], management during the treatment period is essential for cancer control. Minocycline, topical moisturizers, and steroids are administered to prevent severe diseases, and self-care is important [9]. The exact mechanism of EGFR inhibitor-related skin toxicities remains unclear; however, several reports have suggested inflammatory reactions [10–13] and the effects of the treatments on the immune system [14]. There is no difference in the pathogenesis of toxicity between the antibodies or TKIs. Based on the mechanism, topical steroids are administered to prevent or treat EGFR inhibitor-related skin toxicities. If symptoms are severe, treatment postponement or dose reduction is recommended [15–19]. Various management recommendations and guidelines have been proposed [20–35]. However, management recommendations focused on Asian patients are limited [20, 21] and have not been updated in over 5 years. The skin types of Asians differ from those of other races. Therefore, treatment recommendations tailored to Asian patients should be considered. In this study, we aimed to review preventive and therapeutic evidence in Asia and provide suitable management recommendations for EGFR inhibitor-related skin toxicities in Asian patients.

## Materials and methods

**Fig. 1:**
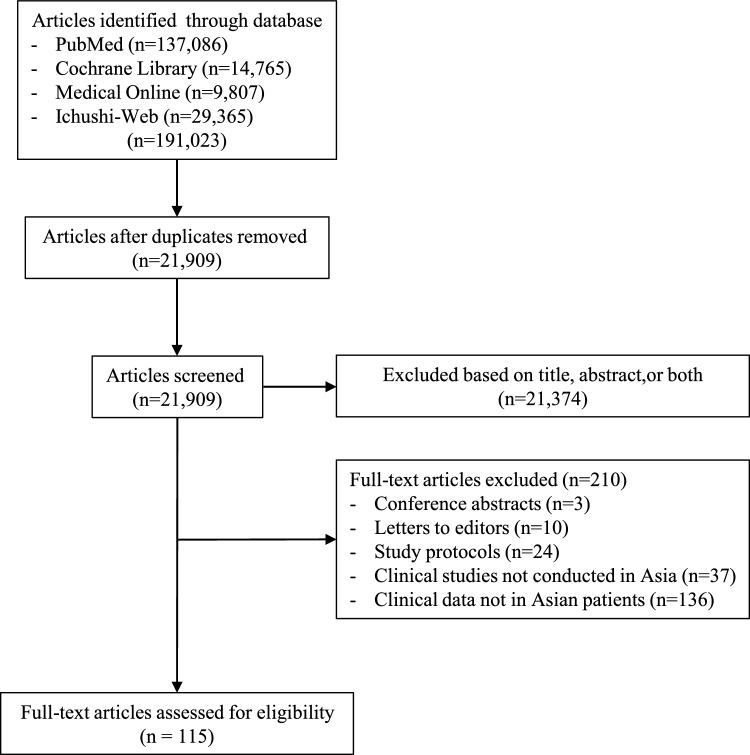
Diagram of study selection/screening process

A total of 191,023 articles were identified by conducting a search on PubMed, Cochrane Library, Medical Online, and Ichushi-Web for articles published from 1928 onwards. Of these, 21,374 publications were excluded after reviewing the titles and abstracts because they were either irrelevant to the research topic or duplicates. In addition, 420 articles were excluded after a thorough review because they lacked usable data. Figure 1 shows the literature screening process. Notably, the number of pertinent studies has been on the rise in recent years.

## Results

### Incidence

EGFR inhibitors cause various skin toxicities. The incidences of acneiform rash, paronychia, and pruritus are relatively high. The incidence of these skin toxicities in Asian patients is described below (Table 1).

**Table 1:** Summary of the incidence of skin toxicities caused by EGFR inhibitors in Asian patients

	Incidence (%)		
	Acneiform rash	Paronychia	Pruritus
EGFR antibodies			
Cetuximab	52–85.1% [36–43]	15–57% [36, 39, 40]	17% [36]
Panitumumab	50–83% [36, 43–45]	11–35% [36, 44, 75]	16–33% [36, 44]
EGFR TKIs			
Gefitinib	11–85.1% [46–56]	6–32.2% [47, 49–51, 54–56]	6–32.2% [47, 49–51, 54–56]
Erlotinib	49–99% [57–61]	4–65% [57–61]	10–42% [57, 58, 60]
Afatinib	18–56.8% [55, 62–65]	18–56.8% [55, 62–65]	10.9–23% [55, 62, 64, 65]
Osimertinib	14.6–59% [66–70]	35–43.9% [67–69]	12–18% [66–70]
Dacomitinib	49–56.5% [54, 56, 57, 71]	21–64.7% [54, 56, 57, 71]	10–20.7% [54, 56, 57]

*EGFR* epidermal growth factor receptor, *TKIs* tyrosine kinase inhibitors

### Acneiform rash

Both EGFR antibodies (cetuximab, 52–85.1% [36–43]; panitumumab, 50–83% [36, 43–45]) and EGFR TKIs (gefitinib, 11–85.1% [46–56]; erlotinib, 49–99% [57–61]; afatinib, 55–89.1% [55] [62–65]; osimertinib, 14.6–59% [66–70]; dacomitinib, 49–56.5% [54, 56, 57, 71]) lead to a high incidence of acneiform rash. The incidence of grade ≥ 3 symptoms is only observed in a small proportion of patients, and these symptoms are rarely life-threatening [72, 73]. However, an acneiform rash can greatly reduce a patient's QOL, including by causing psychological distress. An acneiform rash tends to develop 1–4 weeks after the start of chemotherapy and often persists thereafter [72, 74]. In the early stages of its onset, the skin rash is sterile; however, secondary infections can develop in the chronic phase.

### Paronychia

Paronychia occurs in approximately 50% of Asian patients treated with EGFR antibodies or TKIs. There is no marked difference in incidence between EGFR antibodies and TKIs (cetuximab, 15–57% [36, 39, 40]; panitumumab, 11–35% [36, 44, 75]; gefitinib, 6–32.2% [47, 49–51, 54–56, 75]; erlotinib, 4–65% [57, 58] [59–61]; afatinib, 18–56.8% [55, 62–65]; osimertinib, 35–43.9% [67–69]; dacomitinib, 21–64.7% [54, 56, 57, 71]). Paronychia develops 1–2 months after the start of chemotherapy and occurs in 50% of patients after 6 months [34, 72]. Severe cases of paronychia are often associated with intense pain and require up to 3 weeks for recovery.

### Pruritus

At least 10% of patients receiving EGFR antibodies or TKIs develop pruritus. Severe cases are rare; however, both EGFR antibodies (cetuximab, 17% [36]; panitumumab, 16–33% [36, 44]) and TKIs (gefitinib, 15.6–63% [48–50, 52, 55, 56]; erlotinib, 10–42% [57, 58, 60]; afatinib, 10.9–23% [55, 62, 64, 65]; osimertinib, 12–18% [66–70]; dacomitinib, 10–20.7% [54, 56, 57]) can cause mild pruritus.

### Pathology and differential diagnosis

The pathogenesis of EGFR inhibitor-induced skin rash has not been clearly identified; however, an inflammatory response mediated by chemical mediators has been suggested. EGFR inhibition and the promotion of lipid production in sebaceous glands induce cyclooxygenase-2 (COX-2). Consequently, inflammatory responses mediated by neutrophils and lymphocytes are induced, resulting in skin rash [10–12]. In vitro studies have shown that COX-2 inhibition prevents the production of prostaglandin F2α and prostaglandin E2 in the sebaceous glands [76]. In addition, aspirin inhibits COX-1 and prevents platelet aggregation, thereby suppressing EGFR-TKI-associated skin rash [77]. Therefore, suppression of the inflammatory response is necessary for the management of EGFR inhibitor-induced skin rash. Another possible pathological mechanism is the inhibition of epidermal keratinocyte differentiation [78]. EGFR is normally expressed in epidermal keratinocytes, sebaceous glands, eccrine glands, and hair follicle epithelium. Activation of EGFR normally inhibits the differentiation of basal layer keratinocytes while promoting the terminal differentiation of suprabasal layer epidermal keratinocytes. In addition, EGFR is thought to promote keratinocyte survival [79]. EGFR inhibitors suppress normal keratinocyte differentiation and cause skin toxicity [80].^1^ Pathological hyperkeratinization may occur during the development of inflammatory dermatitis in hair follicles, a process involving acne [81] In particular, there have been multiple reports of the induction of inflammatory reactions [78]. In general, EGFR inhibitor-induced dermatoxicity is sterile; however, *Cutibacterium acnes* may also be a cause [82]. Acne is a known bacterial infection of the sebaceous glands. With regard to differential diagnosis, long-term continuous use of topical steroids, especially on the face, can cause “rosacea-like dermatitis” [83] [84], a side effect that is particularly difficult to distinguish from acne or acneiform rash. Therefore, the continuous use of highly potent topical steroids should be limited to approximately 4 weeks. As it is difficult to distinguish rosacea-like dermatitis from acne vulgaris, which is not related to EGFR inhibitors, the management of EGFR inhibitor-induced skin rash plays an important role during cancer treatment. Unlike hand-foot syndrome (HFS), which is localized to the hands and feet, EGFR inhibitor-induced skin disorders present with systemic symptoms. Cytotoxic anticancer agents and multikinase inhibitors (e.g., capecitabine, taxane, and regorafenib) may cause paronychia in addition to HFS; therefore, the frequency can be increased in regimens that involve the use of EGFR inhibitors with cytotoxic anticancer agents. Pruritus should also be distinguished from other infusion-related reactions. A differential diagnosis may be required if pruritus occurs as an infusion-related reaction to an intravenous injection. Notably, patients with allergic reactions to beef may exhibit an infusion reaction to cetuximab. EGFR inhibitor-induced pruritus rarely occurs during or within 24 h after the administration of chemotherapy, and complaints of generalized pruritus during EFGR inhibitor administration require treatment, assuming that an infusion-related reaction is involved.

### Evaluation of severity

EGFR inhibitor-related skin toxicities are evaluated by the Common Terminology Criteria for Adverse Events (CTCAE) (Table 2) and Dermatology Life Quality Index, and in the case of paronychia, SPOT [85] can be useful for evaluating how the symptoms affect the patient’s QOL. CTCAE is the simplest and most frequently used method; however, it is not suitable for a detailed evaluation.

**Table 2:** Common Terminology Criteria for Adverse Events (CTCAE) v5.0

	Grade 1	Grade 2	Grade 3	Grade 4
Acneiform rash	Papules and/or pustules covering < 10% BSA, which may or may not be associated with symptoms of pruritus or tenderness	Papules and/or pustules covering 10–30% BSA, which may or may not be associated with symptoms of pruritus or tenderness; associated with psychosocial impact; limiting instrumental ADL; papules and/or pustules covering > 30% BSA with or without mild symptoms	Papules and/or pustules covering > 30% BSA with moderate or severe symptoms; limiting self-care ADL; associated with local superinfection with oral antibiotics indicated	Life-threatening consequences; papules and/or pustules covering any % BSA, which may or may not be associated with symptoms of pruritus or tenderness and are associated with extensive superinfection with IV antibiotics indicated
Paronychia	Nail fold edema or erythema; disruption of the cuticle	Local intervention indicated; oral intervention indicated (e.g., antibiotic, antifungal, antiviral); nail fold edema or erythema with pain; associated with discharge or nail plate separation; limiting instrumental ADL	Operative intervention indicated; IV antibiotics indicated; limiting selfcare ADL	–
Pruritus	Mild or localized; topical intervention indicated	Widespread and intermittent; skin changes from scratching (e.g., edema, papulation, excoriations, lichenification, oozing/crusts); oral intervention indicated; limiting instrumental ADL	Widespread and constant; limiting selfcare ADL or sleep; systemic corticosteroid or immunosuppressive therapy indicated	–

*BSA* body surface area, *ADL* activities of daily living

### Medication

#### Prophylactic clinical studies (Table 3)

**Table 3:** Prophylactic clinical studies on EGFR inhibitor-related skin toxicities

Country, year	Type of study	Prophylaxis	Blinding	Population (N)	Chemotherapy	Intervention	Comparator	Outcomes
Japan 2015 [86]	RCT	Minocycline, skin moisturizer, sunscreen, medium class topical steroid	No	N = 95 Metastatic colorectal cancer	Panitumumab	N = 47 Preemptive minocycline 100 mg per day + skin moisturizer, sunscreen, medium-class topical steroid	N = 48 Reactive skin moisturizer	Preemptive measures reduced the incidence of grade ≥ 2 skin toxicities (21.3% vs. 62.5%, P < 0.001)
Japan 2015 [89]	Retrospective study	Minocycline, standard skin care	No	N = 38 Metastatic colorectal cancer	Panitumumab	N = 25 Preemptive minocycline 100 mg per day + standard care	N = 13 Reactive minocycline	Preemptive measures reduced the incidence of grade ≥ 2 skin toxicities (44% vs. 84.6%, P = 0.04)
Japan 2015 [88]	Retrospective study	Minocycline, skin moisturizer	No	N = 96 Advanced pancreatic cancer	Erlotinib	N = 44 Preemptive minocycline 200 mg per day + skin moisturizer	N = 52 Reactive minocycline	Preemptive measures reduced the incidence of any grade acneiform rash and xerosis (47.7% vs. 80.8%, p < 0.001; 2.3% vs. 19.2%, p = 0.01)
Japan 2021 [87]	RCT	Clarithromycin, skin moisturizer, sunscreen	No	N = 150 Metastatic colorectal cancer	Panitumumab	N = 75 Preemptive clarithromycin 200 mg twice per day	N = 75 Reactive clarithromycin (permitted if grade ≥ 2 skin toxicity occurred)	Preemptive measures reduced the incidence of grade ≥ 2 skin toxicities (21% vs. 55%, P < 0.001)
Japan 2023 [91]	Retrospective study	Addition of weak-class topical steroid to systemic minocycline	No	N = 87 Metastatic colorectal cancer	EGFR antibodies	N = 65 Preemptive minocycline 100 mg per day + skin moisturizer and medium-class topical steroid	N = 22 Without preemptive medium-class topical steroid	No significant difference was found in the incidence of overall grade ≥ 2 skin toxicities (56.9% vs. 63.6%, P = 0.93)
Taiwan 2021 [93]	Case control cohort study	Topical betaxolol 0.25% solution	No	N = 131 NSCLC	EGFR TKIs	N = 40 Topical betaxolol 0.25% solution twice a day	N = 91 Without topical betaxolol 0.25% solution	Preemptive measures reduced the cumulative incidence of paronychia (P < 0.01)
Taiwan 2022 [92]	Retrospective study	0.5% timolol ophthalmic solution + neomycin/tyrothricin ointment	No	N = 22 NSCLC	EGFR TKIs	N = 22 0.5% timolol ophthalmic solution + neomycin/tyrothricin ointment	–	Overall, complete, and partial response rates were 83.3%, 18%, and 68%, respectively
Japan 2019 [94]	RCT (self-control)	Adapalene gel 0.1%, skin moisturizer, minocycline	Yes	N = 26 NSCLC or head and neck cancer	EGFR inhibitors	N = 26 Adapalene gel 0.1% + skin moisturizer + minocycline (one side of the face)	–	No significant difference was found in the lesion count (9.8% vs. 12.8%, P = 0.12)
Republic of Korea 2013 [95]	Historical control	Vitamin K1 cream	No	N = 40 Metastatic colorectal cancer	Cetuximab	N = 40 vitamin K1 cream	–	No significant difference was found in the incidence of grade ≥ 2 skin toxicities between the historical control and experimental groups (55.5% vs. 42.5%, P = 0.34)
China 2022 [37]	RCT	Honeysuckle	No	N = 139 Metastatic NSCLC and colorectal cancer	EGFR inhibitors	N = 46 Honeysuckle (10 g honeysuckle in 200 ml soup) orally twice daily	N = 46 Reactive therapy (minocycline, topical clindamycin, and 1% hydrocortisone when grade ≥ 1 acneiform rash occurred)	Preemptive measures reduced the incidence of grade ≥ 2 skin toxicities (21.7% vs. 45.6%, P = 0.027)
Japan 2018 [98]	Retrospective study	NSAIDs	No	N = 49 NSCLC	EGFR TKIs	N = 49 Oral NSAIDs	–	Co-administration of NSAIDs was a preventive factor (P = 0.044)
Japan 2022 [99]	Retrospective study	NSAIDs	No	N = 167 Metastatic colorectal cancer	Panitumumab	N = 59 Oral NSAIDs	N = 108 Without NSAIDs	Co-administration of NSAIDs reduced the incidence of grade ≥ 1 acneiform rash (21% vs. 55%. P < 0.001)

*RCT* randomized control trial, *EGFR* epidermal growth factor receptor, *TKIs* tyrosine kinase inhibitor, *NSAIDs* non-steroidal anti-inflammatory drugs, *NSCLC* non-small-cell lung cancer

##### Systemic tetracyclines and macrolides

Systemic minocycline and clarithromycin reduce grade >2 skin toxicities by 30–40% [86] [87–89]. Because standard measures were co-administered in the preemptive group, moisturization and utilization of sunscreen were necessary to achieve sufficient preventive effects. The duration of administration and concomitant use of steroids are controversial. No treatment-related adverse events were reported in these studies. Doxycycline has also been reported to exert preventive effects [90] (the criteria did not include Asian patients).

##### Topical steroids

No studies have examined the preventive effects of topical steroids alone; the results of one retrospective study [91].^2^ failed to demonstrate the additive effect of a weak-class steroid topical agent. No significant difference was found in the incidence of grade ≥ 2 skin toxicities in the study; however, grade ≥ 2 skin rash was suppressed by adding a topical steroid.

##### Topical beta-blockers

Preventive and therapeutic effects have been reported [92, 93]. However, no prospective studies have been conducted to date.

##### Other topical agents

Studies have evaluated the efficacy of adapalene gel [94], vitamin K1 ointment [95], and honeysuckle therapy [37]. However, no positive results were reported. Vitamin K1 ointment has been reported to be useful, although the report did not include Asian patients [96]. The efficacy of oral retinoids [97] has not been evaluated in Asian patients.

##### Non-steroidal anti-inflammatory drugs (NSAIDs)

Evidence of the preventive effects of NSAIDs against EGFR inhibitor-related skin toxicities is limited to retrospective and single-center studies [98, 99]. These studies reported positive results, suggesting a preventive effect of oral NSAIDs on EGFR inhibitor-related skin toxicities.

#### Therapeutic clinical studies (Table 4)

**Table 4:** Therapeutic clinical studies on EGFR inhibitor-related skin toxicities

Country, year	Type of study	Therapeutics	Blinding	Population (N)	Chemotherapy	Intervention	Comparator	Outcomes
Republic of Korea 2015 [102]	Single arm	Epidermal growth factor ointment (1 ppm concentration)	No	N = 46 NSCLC or pancreatic cancer	Erlotinib	Epidermal growth factor ointment (1 ppm concentration) was administered twice a day in patients with grade ≥ 2 lesions	–	Epidermal growth factor ointment improved CTCAE rating of rash/acne and itching (p < 0.001)
Republic of Korea 2019 [103]	RCT	Epidermal growth factor ointment (1 and 20 ppm concentration)	Yes	N = 26/27 Advanced NSCLC	EGFR inhibitors	N = 26/27 1 or 20 ppm of epidermal growth factor ointment was applied to grade ≥ 2 skin lesions twice daily	N = 27 Placebo	A linear correlation between epidermal growth factor concentrations and responses was observed (p = 0.012)
Japan 2020 [101]	RCT (self-control)	Vitamin K1 ointment	Yes	N = 30 Patients receiving cetuximab or panitumumab developed acneiform eruptions on the face or chest	Cetuximab or panitumumab	N = 30 Vitamin K1 ointment twice a day	–	No significant difference in the number of acneiform eruptions was found between vitamin K1 ointment and placebo (P = 0.069)

*NSCLC* non-small-cell lung cancer, *CTCAE* Common Terminology Criteria for Adverse Events, *EGFR* epidermal growth factor receptor, *TKIs* tyrosine kinase inhibitor

An RCT evaluating the therapeutic effect of a vitamin K1 ointment has been conducted [100, 101]. However, the primary endpoint was negative. The only RCT that showed positive therapeutic efficacy involved an EGF ointment [102, 103].

## Discussion

### Possible management of EGFR inhibitor-related skin toxicities

#### Acneiform rash

Regarding preventive evidence, the prophylactic use of tetracyclines and macrolides exerted remarkable preventive effects against EGFR inhibitor-related skin toxicities. With regard to the prophylactic administration of topical steroids, a number of studies of the prophylactic use of tetracyclines and macrolides added medium-class steroids prophylactically [86, 87, 89]. The additional effect of the prophylactic concomitant use of weak-class topical steroids with minocycline was not demonstrated in all grades of overall skin toxicities [91]. However, grade ≥ 2 skin rash was suppressed by the addition of a topical steroid in a study. The evidence was retrospective; however, the addition of a stronger (medium) class of topical steroid and use of a modified study design may suggest the efficacy of the addition of topical steroids to tetracyclines and macrolides. Notably, the FAEISS study [104] found no difference in the frequency of grade ≥ 2 skin toxicities in patients in the “ranking-down” (RD) group, which was started on a high-potency topical steroid that was serially ranked down, and a “ranking-up” group, which was started on a weak-potency topical steroid that was serially ranked up at exacerbation. However, the frequency of grade ≥ 2 skin toxicities was lower in the RD group after 8 weeks of anticancer therapy. Based on these results, the prophylactic use of steroids should be considered on an individual patient basis. Tetracyclines are less effective when used in combination with drugs containing metals, such as magnesium; therefore, patients should be advised to shift the timing of dosing. In addition, because macrolides increase the blood concentration of EGFR TKIs (gefitinib [105], erlotinib [105], and osimertinib [106]) by inhibiting cytochrome P450 metabolism, the withdrawal of macrolides is recommended when grade ≥3 adverse events develop. Drug selection should consider drug-drug interactions. Regarding the preventive effects of oral NSAIDs on EGFR inhibitor-related skin toxicities, positive results have been reported in a retrospective study. There is a lack of useful evidence on the use of topical beta-blockers and oral retinoids for the prevention of EGFR inhibitor-related skin toxicities in Asia.

For therapeutic management, topical steroids are customarily administered at the onset of symptoms; however, no studies have demonstrated their efficacy. An RCT evaluating the therapeutic effect of a vitamin K1 ointment has been conducted [100, 101]. The primary endpoint was negative; however, there was a reduction in the number of acneiform eruptions resulting from the application of the vitamin K1 ointment, and a redesigned study is warranted. The only RCT that demonstrated positive therapeutic efficacy was conducted using an EGF ointment [102, 103] and demonstrated the efficacy of the topical application of EGF in replenishing the EGF deficiency induced by EGFR inhibitors. Topical products containing EGF are commercially available in the Republic of Korea but have not been marketed as prescription drugs worldwide. Further large-scale studies are required to verify the possibility of their introduction into clinical practice. Dose reduction can be considered after resumption, as a correlation between dose and blood levels has been reported [15–19, 107]. In addition, side effects caused by tetracyclines, macrolides, topical steroids, and drug-drug interactions should be considered. Tetracyclines and macrolides cause vestibular disorders, and concomitant administration of cations (aluminum-, calcium-, or magnesium-containing products) decreases the activity of tetracyclines and macrolides. Therefore, the dosing interval should be at least 1–2 h [108]. Combined therapy with oral antacids (aluminum hydroxide gel) can reduce serum tetracycline levels [109–111]. Topical steroids can lead to the development of atrophic skin [112] and secondary infections. The higher the potency, the higher the risk of side effects, and the unnecessary administration of highly potent topical steroids should be avoided (Table 5).

**Table 5:** Management of acneiform rash

CTCAE Grade	Management	Follow up
Grade 0 prevention	Moisturizing Skin care Systemic minocycline (100 mg/day) for 6 weeks (continued administration as needed) Medium-class topical steroid (on an individual patient basis)	Until development of acneiform rash
Grade 1 treatment Papules and/or pustules covering < 10% BSA, which may or may not be associated with symptoms of pruritus or tenderness	Same dose of chemotherapy should be considered Continued moisturization and skin care Medium-class topical steroid	2 weeks If symptoms worsen, proceed to the next step
Grade 2 treatment Local intervention indicated; oral intervention indicated (e.g., antibiotic, antifungal, antiviral); nail fold edema or erythema with pain; associated with discharge or nail plate separation; limiting instrumental ADL	Same dose of chemotherapy should be considered Strong or very strong class of topical steroid Topical medium-class steroids should be considered for the face Continued moisturization and skin care Consultation with a dermatologist	2 weeks If symptoms worsen or do not improve, proceed to the next step
≥ Grade 3 treatment Papules and/or pustules covering > 30% BSA with moderate or severe symptoms; limiting self-care ADL; associated with local superinfection with oral antibiotics indicated	Treatment discontinuation or postponement should be considered until symptoms improve to Grade 0 or 1 Strongest class topical steroid Topical medium-class steroids should be considered for the face Systemic steroids (p.o. or d.i.v.) Dose reduction of EGFR inhibitor should be considered Systemic antibiotics should be considered, if infectious complications are suspected	2 weeks

*ADL* activities of daily living *BSA* body surface area, *d.i.v.* drip intravenous injection, *EGFR* epidermal growth factor receptor, *p.o.* per os

#### Paronychia

The preventive measures for paronychia are similar to those for acneiform rash. No trial has demonstrated the prophylactic efficacy of treatments apart from tetracyclines and macrolides. Highly potent topical steroid taping and nail removal are often used for pain relief and anti-inflammation; however, there are no studies that can be discussed in this literature review. Therefore, the therapeutic protocol was the same as that used for acneiform rash (Table 6).

**Table 6:** Management of paronychia

CTCAE Grade	Management	Follow up
Grade 0 prevention	Moisturizing Skin care Systemic minocycline (100 mg/day) for 6 weeks (continued administration as needed) Medium-class topical steroid (on an individual patient basis)	Until development of paronychia
Grade 1 treatment Nail fold edema or erythema; disruption of the cuticle	Same dose of chemotherapy should be considered Continued moisturization and skin care Medium-class topical steroid	2 weeks If symptoms worsen, proceed to the next step
Grade 2 treatment Local intervention indicated; oral intervention indicated (e.g., antibiotic, antifungal, antiviral); nail fold edema or erythema with pain; associated with discharge or nail plate separation; limiting instrumental ADL	Same dose of chemotherapy should be considered Strong or very strong class of topical steroid Continued moisturization and skin care Consultation with a dermatologist	2 weeks If symptoms worsen or do not improve, proceed to the next step
Grade 3 treatment Operative intervention indicated; IV antibiotics indicated; limiting selfcare ADL	Treatment discontinuation or postponement should be considered until symptoms improve to Grade 0 or 1 Strongest class topical steroid Dose reduction of EGFR inhibitor should be considered Systemic antibiotics should be considered, if infectious complications are suspected Surgical procedures, such as partial nail removal, should be considered	2 weeks

#### Pruritus

There is no preventive or therapeutic evidence specific to itching, which can be discussed in this review. However, antihistamines and systemic steroids may be effective in severe cases. Protocols for the prevention of acneiform rash can be effective in preventing pruritus (Table 7).

**Table 7:** Management of pruritus

CTCAE Grade	Management	Follow up
Grade 0 prevention	Moisturizing Skin care	Until development of pruritus
Grade 1 treatment Mild or localized; topical intervention indicated	Same dose of chemotherapy should be considered Continued moisturization and skin care Topical or systemic antihistamine Medium-class topical steroid	2 weeks If symptoms worsen, proceed to the next step
Grade 2 treatment Widespread and intermittent; skin changes from scratching (e.g., edema, papulation, excoriations, lichenification, oozing/crusts); oral	Same dose of chemotherapy should be considered Topical or systemic antihistamine Strong or very strong class of topical steroid Topical medium-class steroids should be considered for the face Continued moisturization and skin care Consultation with a dermatologist	2 weeks If symptoms worsen or do not improve, proceed to the next step
Grade 3 treatment Widespread and constant; limiting selfcare ADL or sleep; systemic corticosteroid or immunosuppressive therapy indicated	Treatment discontinuation or postponement should be considered until symptoms improve to Grade 0 or 1 Topical or systemic antihistamine Strongest class of topical steroid Topical medium-class steroids should be considered for the face Dose reduction of EGFR inhibitor should be considered Systemic antibiotics should be considered, if infectious complications are suspected	2 weeks

*ADL* activities of daily living, *EGFR* epidermal growth factor receptor

#### Difference in recommendations between Asia and Europe or the U.S

For grade ≤2 acneiform rash, low/moderate-potency steroids are recommended in Europe and the U.S. [22, 35], while high-potency topical steroids are recommended in Asia [20, 21]. In the case of pruritus, high-potency topical steroids and antihistamines are recommended for grade ≥ 2 symptoms in Europe and the U.S. [22, 35], which is similar to the recommendation in Asia [20, 21]. For paronychia, povidone iodine 2% and topical beta-blockers are recommended in the case of grade ≥ 2 symptoms in Europe and the U.S. [22, 35] but not in Asia due to the lack of evidence in Asian patients. On the other hand, regarding steroid potency, high-potency topical steroids are recommended for grade 1 symptoms in Asia [20, 21] but not in Europe or the U.S. [22, 35] (Table 8). In general, strong topical steroids are recommended for Asians in the early stages of the disease. This recommendation is based on expert opinions rather than evidence-based arguments. Compared to European and American skin, Asian skin has a thinner stratum corneum (outermost layer) and more densely packed cells. This makes Asian skin more sensitive and overreactive to irritation [113, 114] compared to European and American skin. Therefore, early anti-inflammatory treatments may be required. In addition, Asian skin contains more melanin than European and American skin, which creates a natural protective effect against UV radiation [115]. However, European and American skin, which contains less melanin, is more prone to steroid-induced hyperpigmentation. Additionally, as shown by the results of an Asian subset analysis [67] of the FLAURA study [69], the frequency of EGFR inhibitor-related dermatoxicity tends to be higher in Asians. Regarding the onset time of EGFR inhibitor-related skin toxicity, early onset occurs in both Asians and non-Asians [20] [35]. Thus, experts may not recommend the early use of steroids.

**Table 8:** Difference in recommendations between Asia and other countries

	Asia	Europe and the U.S
Grade 2 acneiform rash	High-potency topical steroid [20, 21]	Low/moderate-potency steroid [22, 35]
Grade ≥ 2 pruritus	High-potency topical steroid and antihistamines [20–22, 35]	
Grade ≥ 2 paronychia	Potent topical corticosteroids [21]	Topical povidone iodine 2% and topical beta-blocker [22, 35]
Grade 1 paronychia	High-potency topical steroid [20, 21]	Topical povidone iodine 2% [35]

#### Research questions

A few blinded trials have tested the preventive efficacy. Therefore, it is necessary to demonstrate the efficacy with appropriate blinding. Additionally, evidence is lacking, especially regarding pruritus. However, pruritus can have a severe impact on the patients’ QOL. To our knowledge, no previous studies have focused on pruritus. Therefore, clinical trials with pruritus as an endpoint are warranted.

## Conclusions

In this review, recommendations were derived based on evidence from Asian patients. This review will contribute to the management of EGFR inhibitor-related toxicities in Asian populations.

## Footnotes

1 This reference is of outstanding importance because it shows possible mechanism of lethal skin toxicities induced by EGFR inhibitors and suggest treatment strategies.

2 This reference is of outstanding importance because it shows possibility of additional preventive effect of topical steroid ointment to systemic minocycline against EGFR inhibitor-induced skin toxicities.
